# Transforming dementia care pathway: why shifting to a universal, comprehensive, proactive, local, and integrated model is imperative to improve outcomes

**DOI:** 10.3389/frdem.2025.1719630

**Published:** 2026-01-12

**Authors:** Pallavi Nair, Cornelia Junghans, Michelle Kay, Matthew Harris, Azeem Majeed, Benedict Hayhoe

**Affiliations:** Department of Primary Care and Public Health, School of Public Health, Imperial College London, London, United Kingdom

**Keywords:** dementia, mild cognitive impairment, primary care, community-based care, underserved communities, health system transformation, community health and wellbeing workers, ComPROACT

## Abstract

Dementia care in the United Kingdom (UK) faces vast and complex systemic challenges, impacting not only individuals with dementia but also their families, communities, and the broader healthcare system. We outline these challenges and advocate for a transformative shift towards proactive and integrated community-anchored models of care. We highlight the UK-based Community Health and Wellbeing Workers (CHWW) model, which already delivers universal, holistic household-level support and is uniquely positioned within the communities to address dementia care gaps. Building on this foundation, we introduce ComPROACT, a CHWW-led outreach approach targeting cognitive decline and dementia across the care continuum. This initiative integrates both pre- and post-diagnostic dementia care support, particularly in underserved communities, not by creating new parallel services but by reimagining existing neighbourhood-based roles. Empowering CHWWs to be dementia-capable offers a sustainable pathway to better bridge the systemic gap between primary care, community, and dementia services.

## Introduction

Dementia is one of the most pressing global health challenges, contributing significantly to both disability and dependency among older adults worldwide (Dementia, 2025). In the UK, nearly 1 million people have dementia which is projected to exceed 1.4 million by 2040. The financial burden is expected to rise by over 114% in 2040 (Wittenberg et al., 2019), driven by escalating needs on hospital, primary care, social services, and unpaid carers.

Over the past two decades, substantial advances have been made in dementia care, including introducing national dementia strategies, establishing memory clinics, translating pharmacological research, and early biomarkers studies into practice (NICE, 2018; The Dementia Care Pathway, 2025; Korologou-Linden et al., 2024). Alongside biomedical advances, dementia-friendly initiatives such as Time for Dementia (Daley et al., 2025), Admiral Nurses (Aldridge and Harrison Dening, 2019) programmes, in addition to overall patient and public involvement (PPI) have made important strides (Di Lorito et al., 2024).

However, despite these efforts, for those living with dementia and their families the reality often feels very different. Diagnosis remains delayed or missed altogether (GOV.UK, 2025a). Support is inconsistent, leaving families with limited guidance to shoulder overwhelming burden of care. In this context, burden of care refers to the cumulative psychological, physical, time and financial strain that families experience arising from service fragmentation, limited coordination across health-social care, and costs shifted to households. This framing aligns with World Health Organization (WHO) perspectives on system-level contributors to family strain (World Health Organization, 2025). For people in underserved communities, services are often absent or poorly matched to cultural, social, and linguistic needs (Ighomereho, 2025; Care Quality Commission, 2025a). The paradox is that dementia care has advanced in many respects, yet it continues to fall short of delivering meaningful, continuous, and equitable support.

This position paper argues that while progress has been real, it is crucial we harness it using community support to sustain these effective solutions. The current health system designed to provide support remains largely reactive, fragmented, and unprepared for the scale of what lies ahead (GOV.UK, 2024; Wheatley et al., 2021; Bynum, 2025). Addressing this requires reimagining dementia care not just as a clinical issue but acknowledging the complex and deeply social nature of dementia. Integrated, proactive, and community-led solutions offer a promising approach to bridge some of the persistent gaps in care.

## The human realities of dementia

Beyond the policy targets and statistics lies a deeper, more human story, one of unmet needs, stigma, and systemic neglect. Despite being one of the most feared conditions among people over 55 (Alzheimer’s Association, 2025), dementia remains under-communicated, under-recognised, and under-addressed. Public awareness for dementia lags behind other major health conditions campaigns addressing diabetes, obesity, and cardiovascular disease (GOV.UK, 2025b). This invisibility contributes to widespread misconceptions that dementia is inevitable, untreatable or only a condition of old age. Compounded with fears of losing identity and autonomy, these beliefs intensify the psychological burden, leading to exclusion from care and community support (Alzheimer’s Association, 2025).

Many of these challenges are particularly acute for marginalised and underserved groups (Kenning et al., 2017). Lack of accessible information and mistrust in healthcare (Care Quality Commission, 2025a) lead to reduced help-seeking behaviour, increasing the risk of poor outcomes (Kenning et al., 2017; Giebel et al., 2021). Often the mainstream service models insufficiently address these diverse needs of vulnerable populations. Around 40% of people live alone (Clare et al., 2025), resulting in delayed diagnosis, missed care opportunities, and increased risk of unsafe living conditions and hospitalisation (Dementia, 2025; Alzheimer’s Association, 2025).

People with learning disabilities, sensory impairments, and younger-onset dementia (YOD) frequently face misdiagnosis due to diagnostic overshadowing (Walendzik et al., 2025; Leroi et al., 2022), and have limited access to age-appropriate services largely designed for older population (Giebel et al., 2021). Meanwhile, unpaid carers, often family members and close friends, carry a disproportionate burden of care that is frequently overlooked. They form an invisible workforce, shouldering half of dementia-related costs (Dementia, 2025; Wittenberg et al., 2019) and providing an average of 5 h of care daily (Dementia, 2025; Alzheimer’s Association, 2025). The emotional, physical and financial toll is substantial, with many experiencing burnout, social isolation, and deteriorating health (Nielsen et al., 2021; Willis et al., 2009).

Other underserved communities also face distinct barriers to care. Estimates suggest that around 7% of the dementia population may identify as lesbian, gay, bisexual, or transgender (LGBT) (Dementia, 2025). These individuals experience discrimination, under-diagnosis, and historical trauma contributing to higher rates of social isolation, and health disparities (Liu et al., 2025). Many report that healthcare providers are unaware of their sexual orientation or gender identity, limiting access to culturally competent care (Nowaskie and Sewell, 2021). Similarly, people from Black, Asian, and minority ethnic (BAME) communities face both delayed and under-diagnosis. Culturally inappropriate care leaves many families without accessible or acceptable support (Ighomereho, 2025; Care Quality Commission, 2025a; Kenning et al., 2017). Stigma often silences conversations about memory decline while services rarely adapt to cultural or linguistic needs. Those in the most deprived areas are twice as likely to develop dementia compared with most affluent areas (NHS England, 2024).

Poverty and material deprivation disproportionately exacerbate risk exposure, psychological distress, hinders access to timely care. A recent review highlights that, when viewed through a social health lens, these unmet needs reflect systemic inequities (Koh et al., 2025). Critically, these determinants rarely act in isolation; they intersect across social positions (e.g., poverty, gender, ethnicity, disability), compounding disadvantage and amplifying stigma. This intersectionality deepens exclusion throughout the care pathway (Rai et al., 2020).

While each group faces unique needs across groups, services remain poorly designed for diversity, driving inequity in access, care experience, and outcomes.

## Persistent systemic shortcoming in dementia care

Despite the visibility of dementia within health policy, structural weaknesses persist at every level of the system.

### Weaknesses in diagnosis and inconsistent support

Timely diagnosis is crucial for access to treatment, planning, and support (GOV.UK, 2025a). Diagnostic practices are expensive, time-consuming, and often inadequate, delaying diagnosis, and escalating both suffering and healthcare costs. Complex referral pathways contribute to further delays in diagnosis and access to care (Hayhoe et al., 2016). While emerging advances such as blood-based biomarkers and disease-modifying treatments offer hope (Korologou-Linden et al., 2024), without system-wide reform, these innovations risk being inaccessible to marginalised populations.

There is overwhelming reliance on family carers to identify and escalate needs, with little structured review or coordination from symptom onset to follow-up after initial contact. This lack of continuity means disease progression and changing needs are rarely tracked. Unlike other long-term conditions such as diabetes or Parkinson’s disease, dementia lacks a standardised pathway with routine reviews or a named care coordinator (Abrams et al., 2024). This absence of continuity forces families into a reactive mode, often seeking help only during crises. Many families report a lack of ongoing relational support before or after diagnosis, leaving them isolated in navigating a complex healthcare system (Paladino et al., 2025), This highlights systemic gaps in anticipatory guidance and care planning, reinforcing evidence that dementia services remain geared towards discrete interventions rather than sustained, holistic care (Livingston et al., 2024; Bartels et al., 2024).

While post-diagnostic support remains inconsistent in many settings, with limited proactive follow up (Wheatley et al., 2021; Nielsen et al., 2021; Alzheimer's Society, 2022), emerging programmes illustrate diverse responses to these gaps. In the UK, PriDem embeds clinical dementia leads within primary care networks to improve continuity and integration (Bamford et al., 2023). Similarly, EVIDEM explored evidence-based dementia care from timely diagnosis by developing educational packages for General Practitioners (GP) to end-of-life care interventions highlighting the complexity of translating policy into practice (Iliffe et al., 2015). Alongside, Rare Dementia Support provides tailored, continuous support for rare and young-onset dementia (About RDS, 2025). The NIDUS programme adds a personalised, theory-informed approach, training health and social care professionals to deliver psychosocial strategies to support people with dementia and carers in community settings (Zabihi et al., 2022). Internationally, Australian initiatives such as the Specialist Dementia Care Program (Atee et al., 2025), policy/practice on person-centred models (Butchard and Kinderman, 2019), and realist-informed evaluations of implementation of care homes intervention (Devi et al., 2021) underscore similar responses to fragmentation and workforce strain. These examples collectively reinforce that while systemic shortcomings persist, multiple pathways are being tested to achieve more equitable, integrated dementia care.

### Fragmented and episodic models of care

Even when services are accessed, they are often singular and narrowly targeted, addressing specific deficits, symptoms or stages rather than adopting life-course approaches (Giebel et al., 2021; Livingston et al., 2024; Care Quality Commission, 2025b); such as, memory assessment clinics for diagnosis, long-term medication management in primary care, or crisis response teams often operate in silos, offering discrete interventions, rather than sustained, holistic care. Meanwhile, care coordination roles (e.g., dementia link workers, dementia advisors, dementia care navigators) in the National Health Service (NHS) and through voluntary organisations vary widely in availability and scope with no national standards, exemplify this fragmented approach (NHS England, 2024). Few initiatives like extended GP roles, proactive risk profiling to promote joined-up care through Integrated Care Systems (ICSs) since 2022, are yet to deliver measurable improvements in dementia pathways (The King’s Fund, 2024).

### Commissioning and infrastructure gaps

Health, social care, and voluntary sectors services are funded and delivered separately, with little coordination (The King’s Fund, 2024). For example, memory assessment clinics are commissioned by integrated care boards (ICBs), social support by local authorities, psychosocial or carer support by the voluntary and community sector. As a result, coordination and integration rely on local innovation rather than national standards, continuing postcode inequity (Care Quality Commission, 2025b).

### Mounting NHS pressures

These systemic issues are further complicated by wider pressures that NHS faces today, including rising demand from an ageing population, shortages in specialist staff and prolonged delays between appointments to follow-up (GOV.UK, 2024). The recently announced 2025–26 NHS priorities (Cooper et al., 2016), which shift focus away from dementia, risk placing even greater strain on families, services, and the system.

## Intervention gaps: a system misaligned with need

### Policy ambition versus implementation

Although national strategies increasingly emphasise holistic care (e.g., NHS wellness pathway), implementation remains limited (Cooper et al., 2016). Funding and performance metrics favour short-term, fundamentally reactive interventions triggered only after crises such as hospital admission, wandering, aggressive behaviour or carer burnout (e.g., safeguarding alerts or care package requests) (Care Quality Commission, 2025b). This creates a system allowing problems to accumulate and widen regional inequities across, in service provision and workforce capacity (Dementia, 2025; Care Quality Commission, 2025a).

While our perspective is UK-focused, dementia care strategies across Europe vary widely in post-diagnostic access, care affordability, and legal rights for people with dementia, as shown by European Dementia Monitor (Alzheimer Europe a.s.b.l., 2025). Countries with established national strategies such as the Norway and Finland tend to score higher, but no country excels across all 10 domains (Alzheimer Europe a.s.b.l., 2025). These frameworks rarely address the heterogeneity of ‘*what works for whom*’. Recent comparative review highlights the need for a stronger intersectional and social-health lens in psychosocial recommendations (Neal et al., 2025).

### Prevention: a missed public health opportunity

Despite compelling trial evidence [FINGER study (Rosenberg et al., 2020), WW-FINGER (Kivipelto et al., 2020), and US POINTER] that lifestyle changes can reduce dementia risk, the UK lacks a systematic dementia prevention programme equivalent to the NHS Diabetes Prevention Programme. Public messaging remains insufficient, particularly in midlife when prevention efforts could be most effective (GOV.UK, 2025b). Up to 40% of cases could be delayed or prevented by addressing lifestyle and vascular health. This represents a critical miss to shift from late-stage management to upstream public health initiative.

### Missed opportunities for early intervention

Beyond prevention, opportunities for early clinical interventions are also consistently missed. Mild cognitive impairment (MCI), a recognised transitional stage before developing clinical dementia, affects about 15% of adults aged over 65 (GOV.UK, 2025a), is a key window for timely action. Yet, current National Institute of Clinical Excellence (NICE) guidelines exclude MCI from formal recommendations, citing limited high-quality evidence and inconsistent diagnostic criteria (NICE, 2018). While clinically relevant, this gap leaves clinician without clear guidance and patients without structured support to reduce long-term burden of care, progression to dementia.

High-risk groups face greater urgency. People with learning disabilities (Down’s syndrome) or sensory impairments (Hearing impairments) often develop dementia earlier than general population (NHS England, 2024). Symptoms are frequently missed or misattributed due to diagnostic overshadowing. Similarly, early signs recognised in primary care or community settings rarely trigger timely, proactive support widening the gap between early concern and meaningful action.

### Narrow research and intervention focus

Research and intervention strategies often mirror these systemic shortcomings. Most dementia trials and evaluations remain vertical, meaning disease-driven, targeting discrete symptoms, single domain (e.g., memory, sleep) or single outcome (e.g., delayed care home admission) (Livingston et al., 2024). Few adopt multi-component, relational, or life-course-oriented approaches that reflect the lived realities of dementia (Dam et al., 2016; Kwok et al., 2025). However, research investment has disproportionately favoured biomedical approaches. Breakthroughs in diagnostics and treatment are vital, but have overshadowed equally important questions: How do people already diagnosed with dementia live independently in the diverse communities? What models of care reduce inequity? Which interventions improve quality of life? As a result, the evidence base for integrated, community-based models of care remains under-developed. Populations most at risk of exclusion remain under-represented in research. Without inclusive research, the system continues to design services for a narrow ‘average’ that fails to reflect reality.

A further gap concerns lack of understanding of what works for whom, under which contexts, and how effects are generated in complex and multi-component dementia care. This gap often results in generic, one-size-fits-all interventions. It is closely linked to methodological limitations, as highlighted by the INTERDEM Methodology Taskforce, which advocates for theory-driven, mixed-method approaches (e.g., realist and process evaluations) to unpack mechanisms and context–mechanism–outcome configurations for personalised dementia care (Bartels et al., 2024).

### Primary care: an overstretched anchor

Primary care, the cornerstone of UK healthcare remains chronically under-resourced and ill-equipped to manage the complexity of dementia, weakening the foundation to deliver continuous support (Woolford et al., 2024). GPs face short 10-min consultation times and heavy caseloads (Bradley et al., 2024), limiting their capacity for comprehensive assessment or relational continuity. While support roles such as dementia care navigators exist, provision remains patchy and are typically available only after diagnosis, leaving pre-diagnostic and household needs unmet.

## Consequences: a system that arrives too late

The cumulative effect of these shortcomings is a system that fails patients and carers at multiple stages of the care pathway; missed prevention, late presentation, delayed diagnosis, inconsistent follow-up and growing family strains. Table 1 summarises these challenges highlighting the impact of unmet needs and broader implications for care. This creates a system where problems accumulate invisibly, escalating into costly, traumatic outcomes for families and healthcare system, leaving underserved groups particularly vulnerable. Without bold systemic reform, the dementia care pathway will continue to be defined by fragmentation, inequity, and crisis.

**Table 1:** Unmet needs, systemic challenges, and intervention gaps in dementia care.

Domain	Challenge	Impact	Implication for Care
Lived experience and realities	Stigma and fear	Reluctance to seek help	Hidden prevalence, missed support, families enter care late and in crisis
	Cultural and language barriers	Symptoms misunderstood or normalised	Delayed recognition, underdiagnosis in ethnic minority communities
	Delayed or missed diagnosis	Loss of opportunity for early intervention and planning	Families forced into crisis mode; late entry into care pathways
	Limited carer support and respite	Carers shoulder extensive responsibilities, unsupported	Carer breakdown; early institutionalisation of patients
	Inadequate post-diagnostic support	Families left unsupported after diagnosis	Decline accelerates; carers experience burnout
	Underrepresentation of underserved groups/ ethnic minorities in research	Limited applicability of evidence	Inequitable care models: interventions not trusted or adopted
Systemic challenges	Fragmented care pathways	Lack of continuity across services	Poor health outcomes, increased family strain
	Siloed commissioning	Disjointed and overlapping services; medical and social needs addressed separately	Inefficiency, holistic needs unmet, duplication, inequitable access
	No care infrastructure	No named coordinator	Care navigation responsibilities fall on families, leading to gaps and stress
	NHS pressures	Long waits, staff shortages	Innovations may not reach those in need
	Hospital-centric model	Over-reliance on hospitals and crisis care, reactive interventions dominate	Preventable hospitalisations and higher long-term costs
	Limited culturally sensitive resources	Generic interventions do not meet community needs	Poor engagement and trust; inequitable outcomes
	Inadequate attention to advanced dementia and care planning	Families left without guidance in late stages	Poor quality of life; distress for carers; avoidable suffering
	Lack of neighbourhood-based collaboration	Services remain distant and centralised	Missed opportunities for local, relational support
Intervention gaps	Primary care pressures (time, workforce shortages)	Limited capacity for dementia assessments and reviews	Missed opportunities for proactive care; focus remains episodic
	Short-term funding and pilot-driven models	Innovations not scaled or sustained	Cycle of fragmentation continues; loss of trust in system
	Limited prevention and risk reduction focus	Modifiable risk factors overlooked	Rising prevalence; missed opportunity to delay onset
	Narrow research focus (biomedical over social/relational)	Limited evidence of cultural, and community-based interventions	Policies/guidelines miss non-clinical solutions; undervaluing lived experience
	Missed opportunities in early intervention (MCI, Down’s syndrome)	Critical window for early support ignored, Dementia symptoms missed or misattributed	Families lose chance for timely planning; NICE guidance leaves clinicians unsupported, disproportionately impacting vulnerable groups
	Prevention not embedded in practice	Modifiable risk factors (e.g., vascular, lifestyle) under-addressed	Rising prevalence; lost chance to delay or reduce onset
	Lack of dementia-capable workforce at community level	Frontline workers unprepared to identify or support dementia	Care remains biomedical and episodic; community support underutilised
	Inconsistent adoption of global lessons	Proven task-sharing models underutilised in high-income countries	Missed opportunities for scalable, cost-effective solutions

## Towards community-led models of care

### From systemic gaps to community anchors

The scale and complexity of both pre- and post-diagnostic dementia care calls for a paradigm shift; from hospital-centric, episodic interventions to integrated, proactive, relational and community-driven solutions. The UK Government’s 10-Year Health Plan for England envisions an NHS that “predicts and prevents ill health rather than simply diagnosing and treating it” (GOV.UK, 2025c). This aligns with Lord Darzi’s call for a shift from hospital to community and from treatment to prevention (GOV.UK, 2024).

To transform dementia care at community level, we must acknowledge the potential of neighbourhood-based model of collaborative care to ensure no individual or family faces dementia alone. Embedding dementia capabilities into existing, community-anchored structures offers a more sustainable approach path forward rather creating new siloed resource-intensive services.

### Task-sharing with primary care as a principle

Given the urgency and fragmentation in the current system with escalating costs, solutions must strengthen existing services. Task-sharing with primary care can be cost-effective way forward. Evidence suggests it could reduce dementia costs by up to 40% (Wittenberg et al., 2019). General Practitioners, as the first and most consistent point of contact, remain pivotal to both dementia diagnosis and ongoing care. But empowering frontline workers with dementia-specific culturally informed skills, enables proactive support for both primary and community care networks.

### Learning from global evidence

Community health workers (CHWs) have improved chronic disease care worldwide such as diabetes, HIV, and asthma (Haregu et al., 2024). In low- and middle-income countries CHWs also support dementia care through lifestyle modification, early detection, and better service coverage (Alam et al., 2021). These international models offer scalable solutions that could be adapted to high-income contexts. Despite their potential, CHWs in dementia care remain largely underutilised in countries like the UK and USA (Johnson et al., 2025).

### The UK Community health and wellbeing workers (CHWW) model: tessellating care across primary, secondary care and community

The UK CHWW model represents a structured adaptation of international community health worker programmes, most notably inspired from Brazil’s Family Health Strategy (FHS) (Harris, 2012; Harris and Haines, 2012; Junghans et al., 2022; Macinko and Harris, 2015). Since its inception in1994, the FHS has evolved into one of the world’s largest community-based primary care systems, currently reaching over 80% of Brazil’s population and demonstrating sustained improvements in equity, chronic disease management, and reduction in avoidable hospitalisations (Ministério da Saúde, 2025).

Introduced in 2021, the UK CHWW model offers a frugal, rapidly scalable, and effective response to some of the most pressing challenges confronting healthcare today (Junghans et al., 2024). CHWWs are recruited from their neighbourhoods and conduct proactive monthly household visits, enabling early identification of health and social care needs before they escalate into crises. Developed on the CHU-I principles (Junghans et al., 2023) (Table 2): Comprehensive, Hyperlocal, Universal, and Integrated; CHWWs serve as a bridge between communities and primary care representing the Community and Primary Care infrastructure.

**Table 2:** Overview of the CHU-I principles.

**Principles**	**Description**	**Implications for dementia care**
**Comprehensive**	Life-course approach, not defined by age, clinical need or risk profile, providing light touch bio-psycho-social support for all ages and all residents, with a focus on prevention, health promotion and service navigation. Integrating social networks and communities.	CHWWs have a mandate to support lifestyle modifications for all members of the household, support informal carers, spot cognitive changes early and connect people in the community in a personalised and culturally sensitive manner, providing longitudinal input.
**Hyperlocal**	Geographical, covering well-defined neighbourhoods or postcodes, of between 100-150 households per CHWW. CHWWs are recruited from the local area, have connections to or are very familiar with that area, live within walking distance from where they work.	CHWWs have proximity to local language and culture and intimate knowledge of professionals and services in the locality.
**Universal**	All households within the geographical area are visited once per month, or more frequently if there is a need. No referral, no discharge unless residents move out of the area, all residents’ needs are supported.	There is no need for someone to descend into crisis first or even recognise that there is an issue. The proactive approach enables CHWWs to spot cognitive changes or a carer struggling even before the family does.
**Integrated**	Paid and trained, with NHS honorary contracts, NHS email, and access to electronic patient records (EPRs) after appropriate training and approvals. Participate in regular multidisciplinary teams (MDTs). Clear links into Voluntary and Community Sector (VCS) organizations, local authorities and allied health and social care professionals. Identified CHWW on patient record and defined geographies with CHWW contact details available to all staff.	CHWWs are ideally placed to bring in GPs, care navigators, community matrons, social care and other system services as appropriate, as well as connect with the appropriate local services.

The CHWW model was built on an initial programme theory (IPT), developed through iterative qualitative analysis to explain how and under what conditions neighbourhood-based, universal outreach roles improve access, trust, and continuity, particularly in underserved, multi-ethnic areas (Junghans et al., 2023).

By delivering long-term support without discharge, CHWWs reduce fragmentation and extend the reach of traditional, referral-based care. Early pilot in Westminster demonstrated feasibility and strong community acceptance, particularly among underserved populations (Junghans et al., 2023). Current expansion to 25 localities across the UK, including six London boroughs, reflects growing national appeal. A modelling study estimated that scaling up the CHW workforce nationally, integrated within primary care and based on England’s GP-registered population, would require approximately 110,585 CHWs. The projected annual cost of this scale-up was £2.22 billion, representing 1.3–1.8% of total NHS expenditure, depending on the annual budget baseline used (Hayhoe et al., 2018). This national rollout could yield substantial returns by connecting services, shifting the focus to prevention, and embedding care within trusted neighbourhood networks.

While the CHWW model shows promise, key implementation challenges must be addressed to ensure its sustainability and scalability. Funding instability is a major concern; short-term cycles can undermine long-term impact, with programmes often discontinued before benefits are fully realised (Junghans et al., 2023). Incomplete NHS integration (CHWWs are not yet embedded as NHS professionals), and onboarding hurdles limit continuity and data sharing (Riley, 2025). Role clarity and overlap with existing services (e.g., social prescribing) require local alignment (Tredinnick-Rowe et al., 2024). Ambitious patch sizes, and variable commissioning frameworks further challenge workforce resilience and evaluation consistency (Riley, 2025).

Despite these challenges, the model has recently been recognised in national policy. The 10-Year Health Plan explicitly highlights their role as a scalable, neighbourhood-based solution particularly in underserved areas (p. 33) (GOV.UK, 2025c). Their inclusion signals growing institutional support for embedding such roles into the future of integrated, community-led care. This approach signals a paradigm shift towards a community-anchored, place-based model, that integrates medical and psychosocial care.

### CHWWs as a dementia-capable workforce alongside their universal role

CHWWs represent an untapped, hyperlocal workforce for dementia care, ideally positioned to support households directly. They mirror the multi-ethnic fabric of the communities they serve. With tailored dementia training, CHWWs can bridge equity gaps and provide proactive, culturally responsive support across the care continuum. Their hyperlocal presence enables them to bridge some of the persistent gaps across the fragmented system.

By embedding dementia care approaches into CHWW’s universal role, they can offer holistic and sustained support across the NHS Dementia Care Pathway. Global scoping reviews show that integrating dementia-capable competencies within existing CHW programmes enhances awareness, early concern recognition, and continuity in underserved communities (Alam et al., 2021; Johnson et al., 2025). Their trusted relationships built through shared locality, cultural affinity, and lived experience position them to foster open dialogue about memory concerns, identify early cognitive changes and encourage timely help-seeking (Abrams et al., 2024; Junghans et al., 2023). This is particularly effective in-home settings, where regular monthly check-ins create a safe space for concerns to be disclosed more easily than during brief healthcare consultations. Qualitative reports highlight CHWWs role in engaging socially isolated individuals with dementia and multimorbidity who would otherwise have limited access to services (RCGP, 2025), demonstrating relational continuity as a mechanism for bridging structural gaps (Abrams et al., 2024).

Evidence from the Westminster CHWW pilot shows that households receiving at least one CHWW visit had an 82% uptake of cancer screenings and NHS Health Checks compared to those who had not yet received a visit, demonstrating their reach into underserved groups often missed by traditional services (Junghans et al., 2023). Across North-West London, over 100 CHWWs now support more than 20,000 residents, illustrating significant reach across deprived and ethnically diverse communities (NIHR, 2025). In the pre-diagnosis phase, CHWWs can effectively raise awareness through their community engagements, promote brain health message from FINGER study (Rosenberg et al., 2020) and apply Geoffrey Roses’s prevention paradox on proportionate universalism principle that means to tailor support for high-risk groups while serving all (Rose, 1985).

CHWWs integration into existing primary care setting, including access to electronic patient records and participation with multidisciplinary teams, enables seamless coordination and compliance of services. By working closely with carers, GPs and Memory Clinic staff, they can aid in navigating diagnosis and ensuring timely access to support services. During post-diagnostic phase, CHWWs can ensure increase in service access and provide continuity of care with ‘no discharge’ policy, crucial for people with dementia especially those living alone.

By enabling CHWWs to be dementia-capable within their existing universal role avoids creating new specialist silos and instead enables continuity of care and coordination with already existing dementia specialist services. This flexible, place-based approach has the potential to improve outcomes, reduce system demand, and address social health inequalities from prevention until the end-of-life care.

However, a potential limitation of using CHWWs as a dementia-capable workforce is the risk of overwhelming CHWWs who already have broad responsibilities. It is tempting to leverage their universal, regular outreach for multiple initiatives. If unchecked this could lead to workforce burnout and diminish the quality of their relationships with residents. Adequate supervision and safeguarding of workload are therefore essential. CHWWs can only be successful in this role when they have the ability to draw on a wide range of professional support to bring in the technical expertise CHWWs do not possess, for example admiral nurses, palliative care, social care, underscoring the need for integration and cross-sectorial support.

### The way forward: empowering communities and embedding dementia sensitivity

Evidence shows community workers may initially have limited understanding of dementia (Fernandes et al., 2010), but targeted training can significantly improve their knowledge and caregiving capabilities (Reinschmidt et al., 2023). Building on this, we developed ComPROACT (Community Health and Wellbeing Workers led PRoactive Outreach Approaches to Cognitive Decline and Dementia in the CommuniTy), a community-driven multifaceted programme offering tailored dementia training, a toolkit and fostering collaboration with existing services to enhance culturally informed dementia care across pre- and post-diagnostic phases.

ComPROACT is co-designed with people with dementia, careers, healthcare professionals, community organisations, voluntary sectors, residents over 45, and faith leaders. Drawing on international learnings from Brazil and stakeholder inputs, the initiative builds a collaborative, evidence-based framework for a responsive dementia workforce.

ComPROACT prioritises tailored care to underserved communities and groups, promoting earlier identification and culturally informed support to prevent the risk of people falling through the systemic cracks. By leveraging dementia capabilities into CHWW’s universal role, they can become the linchpin of a resilient, cost-effective, community-based dementia system. This innovative, locally embedded approach strengthens existing services rather than creating parallel systems.

However, a potential limitation in implementing ComPROACT is ensuring that design, content, and duration of dementia training remain appropriate for CHWWs, given the breadth of their responsibilities. While brief, skills-based interactive sessions can build confidence and core competencies (Reinschmidt et al., 2025), their impact depends on ongoing supervision, opportunities to learn from people with lived experience, and support from wide range of professionals when task-sharing is required. The aim is to make CHWWs dementia-capable, not dementia specialists. Embedding dementia sensitivity within everyday CHWW practice will therefore require clear role boundaries complementing present dementia specialist, alignment with local priorities, and monitoring of feasibility and outcomes. These considerations will inform the iterative refinement of ComPROACT as implementation progresses.

## Conclusion

At this critical moment of NHS restructuring and strain, models like CHWW are not merely an innovation but a necessity. By shifting the front door of primary care into the community, CHWWs redefine where and how care begins, embedding continuity and equity at the household level. Within this model, ComPROACT programme could offer a transformative blueprint for re-anchoring dementia care locally by prioritising prevention, timely recognition, and culturally responsive relational support across the care pathway. Such approaches have the potential to be particularly vital for underserved communities, where trust and continuity are essential for engagement. Investing in human capital is ultimately an investment in the foundations of trust, sustained engagement, and system resilience. Alongside scientific and technological advances, the future of dementia care depends on integrated community-anchored solutions that proactively address unmet needs before they become unmanageable.

## Data Availability

The original contributions presented in the study are included in the article/supplementary material, further inquiries can be directed to the corresponding author/s.
